# A Branched Biosynthetic Pathway Is Involved in Production of Roquefortine and Related Compounds in *Penicillium chrysogenum*

**DOI:** 10.1371/journal.pone.0065328

**Published:** 2013-06-12

**Authors:** Hazrat Ali, Marco I. Ries, Jeroen G. Nijland, Peter P. Lankhorst, Thomas Hankemeier, Roel A. L. Bovenberg, Rob J. Vreeken, Arnold J. M. Driessen

**Affiliations:** 1 Molecular Microbiology, Groningen Biomolecular Sciences and Biotechnology Institute, Zernike Institute for Advanced Materials, University of Groningen, Groningen, The Netherlands; 2 Kluyver Centre for Genomics of Industrial Fermentations, Delft, The Netherlands; 3 Division of Analytical Biosciences, Leiden/Amsterdam Center for Drug Research, Leiden University, Leiden, The Netherlands; 4 DSM Biotechnology Center, Delft, The Netherlands; 5 Netherlands Metabolomics Centre, Leiden University, Leiden, The Netherlands; 6 Synthetic Biology and Cell Engineering, Groningen Biomolecular Sciences and Biotechnology Institute, University of Groningen, Groningen, The Netherlands; Technical University of Denmark, Denmark

## Abstract

Profiling and structural elucidation of secondary metabolites produced by the filamentous fungus *Penicillium chrysogenum* and derived deletion strains were used to identify the various metabolites and enzymatic steps belonging to the roquefortine/meleagrin pathway. Major abundant metabolites of this pathway were identified as histidyltryptophanyldiketopiperazine (HTD), dehydrohistidyltryptophanyldi-ketopiperazine (DHTD), roquefortine D, roquefortine C, glandicoline A, glandicoline B and meleagrin. Specific genes could be assigned to each enzymatic reaction step. The nonribosomal peptide synthetase RoqA accepts L-histidine and L-tryptophan as substrates leading to the production of the diketopiperazine HTD. DHTD, previously suggested to be a degradation product of roquefortine C, was found to be derived from HTD involving the cytochrome P450 oxidoreductase RoqR. The dimethylallyltryptophan synthetase RoqD prenylates both HTD and DHTD yielding directly the products roquefortine D and roquefortine C without the synthesis of a previously suggested intermediate and the involvement of RoqM. This leads to a branch in the otherwise linear pathway. Roquefortine C is subsequently converted into glandicoline B with glandicoline A as intermediates, involving two monooxygenases (RoqM and RoqO) which were mixed up in an earlier attempt to elucidate the biosynthetic pathway. Eventually, meleagrin is produced from glandicoline B involving a methyltransferase (RoqN). It is concluded that roquefortine C and meleagrin are derived from a branched biosynthetic pathway.

## Introduction

Fungi produce a variety of secondary metabolites, which have diverse activities, ranging from natural antibiotics to simple toxins or immunosuppressants [1]. Most of these metabolites are synthesized either by nonribosomal peptide synthetases (NRPS) or polyketide synthases (PKS) and can be further modified by a range of biosynthetic enzymes. The genes encoding the NRPS or PKS and the modifying enzymes are usually located in the same genetic region and are often co-expressed [2], [3]. Several methods have been developed to identify the secondary metabolite biosynthetic gene clusters and their respective products. Key methods are heterologous gene expression, bioinformatics identification of gene clusters by methods such as SMURF [4] and genome mining strategies in which core synthetase genes are deleted and changes in metabolite production are identified by comparative metabolite profiling [5].

Secondary metabolites genes and/or their clusters were frequently transferred from one organism to another during evolution [6], [7]. Also different organisms may produce similar metabolites [8]. Various fungal species were reported to produce diketopiperazines, a class of pharmaceutically important naturally produced secondary metabolites [9], [10]. Roquefortine C, a diketopiperazine that was first isolated from *Penicillium roquefortine* [11] has now been reported from 25 different *Penicillium* species [12]. It displays bacteriostatic activity against Gram-positive bacteria [13]. Although the exact mechanism of action is not known it appears to interact with cytochrome p450 and interferes with RNA synthesis [14], [15]. Roquefortine C also shows neurotoxic activity in mice and is considered as a contaminant in blue cheese [16], [17]. Meleagrin, a downstream product of roquefortine C, has been proposed to be the precursor of neoxaline, a compound with antimicrobial activity [18].

The filamentous fungus *P. chrysogenum* has been explored for more than eighty years for its excellent fermentation capacity and penicillin production [19], [20]. In recent years, genome sequencing, as well as microarray analysis in combination with genetic modification of *P. chrysogenum,* has provided a strong base to elucidate the role of the secondary metabolite clusters found in this organism. The genome of *P. chrysogenum* encodes 20 putative PKS and 11 NRPS genes including the gene encoding δ-(L-α-aminoadipyl)-L-cysteinyl-D-valine synthetase involved in penicillin biosynthesis [21]. This allowed us to identify the corresponding biosynthetic genes of the roquefortine/meleagrin pathway using a gene deletion strategy as well as quantitative metabolite profiling. Here, we revisit an earlier proposal for a linear pathway for the biosynthesis of roquefortine C that included several ambiguities as well speculations because of incomplete metabolite profiling and enzyme miss assignment. We have elucidated the entire biosynthetic pathway up to meleagrin production including a series of precursors that were structurally analyzed. It is demonstrated that meleagrin is derived from a branched pathway with two biologically derived diketopiperazines as major intermediates.

## Materials and Methods

### A. Chemicals

HPLC-grade acetonitrile and methanol were purchased from Biosolve (the Netherlands). Dichloromethane, formic acid the internal standards reserpine, ranitidine and ampicillin were acquired from Sigma-Aldrich (St. Louis, MO). Meleagrin and neoxaline were purchased from Bio-Connect (the Netherlands). Roquefortine C was obtained from Bioaustralis (Australia). L-Tryptophan, L-Histidine and Mevalonic acid Lactone were purchased from Sigma-Aldrich. All purchased compounds were of highest available purity.

### B. Host Strains, Media, Grown Condition and Plasmid Construction

*P. chrysogenum* strain DS54555, which lacks penicillin cluster genes and Ku70 gene was used as a host strain for deletion analysis and was kindly supplied by DSM Anti-infective. All the strains were grown on YGG-medium [22] for protoplasts formation and transformation. For analysis, cells were grown on SMP medium (glucose, 5.0 g/l; lactose, 75 g/l; urea, 4.0 g/l; Na_2_SO_4_, 4.0 g/l; CH_3_COONH_4_, 5.0 g/l; K_2_HPO_4_, 2.12 g/l; KH_2_PO_4_, 5.1 g/l) for secondary metabolites production using a shaking incubator at 200 rpm for 168 hours at 25°C. Deletion plasmids were constructed by amplifying the flanking regions of the targeted gene with the Multisite Gateway®Three-Fragment Vector Construction Kit (Invitrogen). *Escherichia coli* DH5α was used as host strain for high frequency transformation and plasmid DNA amplification [23]. Tryptophan, histidine and mevalonic acid lactone were dissolve in the same phosphate buffer as used in the culture medium.

### C. Transformation Procedure

Deletion plasmids were transformed to the protoplasts of *P. chrysogenum* strain DS54555 [24]. The phleomycin resistance gene was used as selection marker for the deletion of *roqA, roqT* and *roqD* while acetamidase gene (*amdS*) was used as selection marker for the deletion of *roqM, roqO, roqN* and *roqR* using acetamide as the only nitrogen source for selection [22], [25].

### D. Genomic DNA Extraction, Total RNA Extraction and cDNA Amplification

Genomic DNA (gDNA) was isolated after 96 hours of growth on SMP medium using the modified yeast gDNA isolation protocol [26] in which the fungal mycelium is broken in a FastPrep FP120 system (Qbiogene). Isolated gDNA was measured using a NanoDrop ND-1000 and 5 µg was used for southern hybridization. Total RNA of the host strain was isolated after 168 hours of growth in SMP medium using Trizol (Invitrogen), with additional DNase treatment using the Turbo DNA-free kit (Ambion). Total RNA was measured with the NanoDrop ND-1000 and a concentration of 500 ng per cDNA reaction was used. cDNA was synthesized using the iScript cDNA synthesis kit (Bio-Rad) in a 10-µl end volume.

### E. Southern Blot Confirmation

gDNA of the host and various deletion strains was isolated using the E.Z.N.A. Fungal DNA kit (Omega Bio-tek). Southern blotting was carried out by digesting gDNA (5 µg) with the indicated restriction enzymes. Digested DNA fragments were separated on a 0.8% agarose gel and blotted onto a Zeta-Probe membrane (Biorad) described earlier [27], and hybridized with the indicated probes that were DIG labeled.

### F. qPCR Analysis

The primers used to analyze the expression of all the genes in the Roquefortine/Meleagrin biosynthetic gene cluster i.e. Pc21g15480 (*roqA*), Pc21g15420 (*roqT*), Pc21g15430 (*roqD*), Pc21g15440 (*roqT*), Pc21g15450 (*roqN*), Pc21g15460 (*roqO*) and Pc21g15470 (*roqR*) were designed around an intron to avoid amplification on gDNA (Table S3). For expression analyses, the γ-actin gene was used as a control for normalization. A negative reverse transcriptase (RT) control was used to determine the gDNA contamination in isolated total RNA. The expression levels were determined in triplicate with a MiniOpticon system (Bio-Rad) using the Bio-Rad CFX manager software, with in which the threshold cycle (*CT*) values were determined automatically by regression. The SensiMix SYBR mix (Bioline) was used as a master mix for qPCR with 0.4 µM primers. The following thermocycler conditions were used: 95°C for 10 min, followed by 40 cycles of 95°C for 15 s, 60°C for 30 s, and 72°C for 30 s. Subsequently, a melting curve was generated to determine the specificity of the qPCRs.

### G. Microarray Methods

Triplicate shake flask cultivations were performed with the *P. chrysogenum* strain DS17690 to prepare a proprietary DNA microarray, using the Affymetrix Custom GeneChip program (Affymetrix): After 90 hours of cultivation, samples from shake flask cultures were filtered within seconds and quenched in liquid nitrogen. A standard protocol using Trizol reagent (Invitrogen) and acid phenol-chloroform was used to extract the total RNA followed by cDNA synthesis and cRNA synthesis. Hybridized arrays were scanned and analyzed using the Affymetrix GeneChip Operating Software (GCOS, Affymetrix) as described earlier by van der Berg et al. [21].

### H. HPLC-MS Validation and Analysis

#### 1. Sample preparation

All strains used for gene assignments were grown in quintuplicate according to the procedure described above. Samples for determination of growth curves were grown in eight replicates whereas feeding experiment samples were grown in quadruplicate. Up to 50 µL of a thawed fermentation broth, 8 µL internal standard mixture containing 855 nmol/mL ranitidine, 657 nmol/mL reserpine and 1144 nmol/mL ampicillin was added. Subsequently, 230 µL of methanol was added for protein precipitation and vortexed for 10 minutes. The sample was then centrifuged at 14,000 g for 10 minutes at 10°C. 100 µL supernatant was transferred to an Eppendorf vial and evaporated for 30 minutes in a Thermo-Speedvac (Thermo Scientific, San Jose, CA). The dried sample was redissolved in 100 µL water containing 2% acetonitrile, vortexed for 10 minutes and transferred to an autosampler vial.

#### 2. Reversed-Phase LC-MS

For separation, an Agilent 1200 Capillary pump (Agilent, Santa Clara, CA) coupled to a Surveyor PDA detector (Thermo Scientific, San Jose, CA) and LTQ-FT Ultra mass spectrometer (Thermo Scientific, San Jose, CA) were used. A sample of 5 µL was injected onto a Waters Atlantis T3 column (2.1×100 mm, 3 µm) (Waters, Milford, MA). The elution was performed with a linear gradient starting with 98% of solvent A (1% acetonitrile and 0.1% formic acid in water) and 2% solvent B (1% water and 0.1% formic acid in acetonitrile) for 1.5 minutes at a flow rate of 300 µL/min. The first linear gradient reached 40% B at 22 minutes, the second 100% B at 25 minutes. The column was flushed for 10 minutes at 100% B followed by equilibration for 8 minutes at 100% A. The column effluent was directed to the ESI-LTQ-FT Ultra MS, operated in full scan (m/z 100–2000) in pos/neg switching mode with following settings: Positive ion mode (4 kV source voltage, 14 V capillary voltage, 65 V tube lens), negative ion mode (3 kV source voltage, −18 V capillary voltage, −85 V tube lens) with capillary temperature 275°C, sheath gas flow 50 and auxiliary gas flow 2.

#### 3. Data processing

Raw files were sliced into UV-trace, positive and negative mass trace and subsequently converted into NetCDF, using an in-house tool programmed in MATLAB (MathWorks, Natick, MA). NetCDF files with same polarity were subsequently deconvoluted in DataAnalysis 4.0 (Bruker Daltonik, Bremen, Germany) using the dissect function which was controlled through a macro. The resulting peak tables for each sample were combined and repeating features removed. The combined feature table was used as target list in which each feature was integrated in every individual sample. Samples were grouped regarding their biological origin and statistical tests were performed for determination of significant differences. Discovered features were selected and transferred to LCquan v2.6 (Thermo Scientific, San Jose, CA) for more accurate integration. Peaks were auto-integrated using base peak trace in a mass range of 10 ppm and retention time window of 60 seconds and manually corrected if necessary.

### I. Secondary Metabolite Identification

The identity of **7** and **4** was confirmed by comparing retention time and HR-MS^n^ spectra of samples to commercially available standards. The structure of **1** was determined by Mass Spectrometry (MS^2^ fragmentation and fragmentation tree comparison) as will be detailed elsewhere which was adapted from the metabolite identification pipeline developed with the Netherlands Metabolomics Centre [28], [29].

Compound **3**, **2** and **6** were identified by NMR. **3** and **6** were extracted based on a modified method of Ohmomo [30]. A *P. chrysogenum* culture filtrate was made alkaline with 25% ammonium hydroxide (pH 10) and extracted with dichloromethane. The alkaline dichloromethane layer was evaporated to dryness, redissolved in water containing 50% acetonitrile, vortexed, centrifuged and transferred to an autosampler vial for fraction collection via preparative reversed phase LC on an Atlantis T3 column (10×100 mm, 5 µm ) (Waters Milford, MA). Compound **2** was extracted following the isolation procedure above except using ethylacetate as extraction solvent instead of dichloromethane.

NMR spectra were recorded on a Bruker Avance III 700 MHz NMR spectrometer, equipped with a 5 mm TCI probe. Samples were dissolved in 0.6 mL DMSO/CDCl_3_ 50/50 and acquired at 280 K (**2** and **3**) and 320 K (**6**).

## Results

### Identification of Roquefortine Biosynthetic Gene Cluster

**Figure 1:**
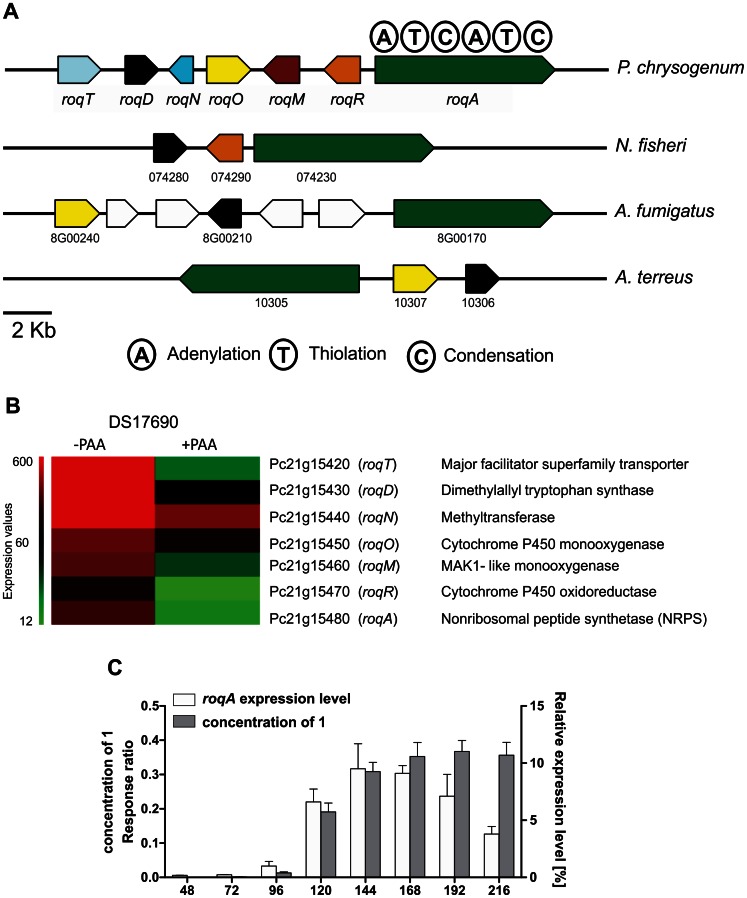
Organization of the roquefortine/meleagrin biosynthetic gene cluster and transcriptomic analysis. (A) Roquefortine/meleagrin biosynthetic gene cluster and their orthologs in phylogenetically relative species. Homologous proteins are indicated with the same color. (B) Microarray analysis of the roquefortine biosynthetic genes in *P. chrysogenum* DS54555 using shake flask culture conditions in the absence (−) or presence (+) phenylacetic acid (PAA). (C) Correlation between the expression level of *roqA* and the concentration of the product HTD (**1)** present in the growth media. The concentration of **1** was determined by HPLC-UV-MS.

In order to identify the secondary metabolite biosynthetic genes under shake flask culture conditions, DNA microarray analysis was performed on the high penicillin yielding *P. chrysogenum* strain DS17690 grown in the presence and absence of the precursor phenylacetic acid (PAA). Most of the secondary metabolite gene clusters were not expressed or only at low levels [21]. However, high levels of expression were observed in the absence of PAA for a gene cluster including the nonribosomal peptide synthetase PC21g15480 (*roqA*), and associated genes Pc21g15420 (*roqT*), Pc21g15430 (*roqD*), Pc21g15440 (*roqN*), Pc21g15450 (*roqO*), Pc21g15460 (*roqM*) and Pc21g15470 (*roqR*) (Figure 1A). These genes were all down regulated when the cells were grown in the presence of PAA and hence are considered as co-regulated in the genome (Figure 1B). For the remainder of the study these genes were abbreviated as *roq* genes because of their involvement in roquefortine production.

### Untargeted Secondary Metabolite Profiling by HPLC-UV-MS

A robust and sensitive quantitative platform for profiling of secondary fungal metabolites from culture broth was developed, validated and applied. As the majority of these compounds are unknown and often present at low concentrations only, the method should be sensitive and provide a high degree of versatility. Herein, we used a high resolution HPLC-UV-MS method with positive/negative ionization switching in combination with UV to allow for the detection of several thousand features. To extract these features from the acquired data, a combination of commercial software packages and in-house scripts were used for untargeted peak discovery and automatic peak integration. By applying a deconvolution algorithm, fragments, adducts and cluster ions as well as their isotopes were grouped and altogether represented mainly by the most abundant ion in the resulting target list which facilitated further data analysis.

The method was validated in which several analytical performance characteristics were determined, but here merely the most important outcomes are reported. As secondary metabolites from the roquefortine/meleagrin pathway showed much higher ionization efficiency in the positive ion mode, only this polarity is described here. For the determination of linear dynamic range, sensitivity and reproducibility, retention times and signal intensities were evaluated for the used (internal) standards at multiple concentrations. These standards comprised of commercially available compounds from the meleagrin/neoxaline pathway (neoxaline, meleagrin and roquefortine C) and a non-related non-endogenous compound (ranitidine), which was used as internal standard. Retention time variations for the standards and several “unknown” endogenous compounds, which are spread over the entire chromatogram of 35 minutes, were limited to maximal 7 seconds (500 injections over 2 weeks of time). The method proved to be linear for each of the standards over at least 3 orders of magnitude (R^2^ ranged from 0.998–0.999 in both, academic and spiked matrix samples) in the appropriate concentration range. Associated Limit of Detections (LOD’s) were determined from internal standard corrected calibration lines (8 levels) and ranged from 3–248 nM, depending on the compound of interest. Due to the absence of endogenous material in the *roqA* deletion strain used for validation, absolute quantitation at the reported low levels is realistic.

The recovery of the method was determined primarily by the extraction of the spiked compounds from the matrix, i.e., fermentation broth. Recoveries ranged from 88% (rsd 7.8%) to 55% (rsd 2.9%) depending on the compound. However, reproducibility measurements showed that both within day and between day reproducibility are well below 15%. In summary, the analytical profiling platform developed here is characterized by good reproducibility and linearity, high coverage and high sensitivity reaching nanomolar levels.

### Metabolite Profiling of the Culture Broth of a *P. chrysogenum* Strain with a Deletion of the Nonribosomal Peptide Synthetase Gene *roqA*

**Figure 2:**
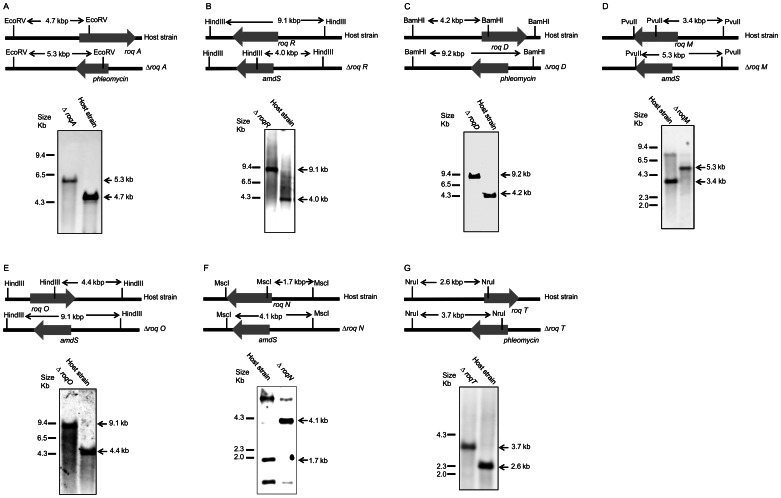
Southern blot analysis for deletion of the genes in the roquefortine/meleagrin pathway. Southern blot hybridization was performed with total DNA extracted from *P. chrysogenum* DS54555 strains with a deletion of the following genes: *roqA* (A), *roqR* (B), *roqD* (C), *roqM* (D), *roqO* (E), *roqN* (F) and *roqT* (G). The DNA was digested with the restriction enzymes as indicated in the schemes.

**Figure 3:**
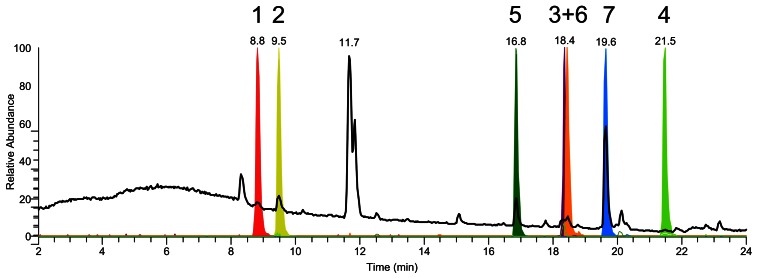
Total ion chromatogram for culture broth of *P. chrysogenum*. Total ion chromatogram (TIC, black) and normalized extracted ion chromatograms (EIC, colored) of secondary metabolites from the roquefortine/meleagrin pathway in the culture broth of *P. chrysogenum* DS54555. HTD (**1**, 8.8 min), DHTD (**2**, 9.4 min), glandicoline A (**5**, 16.8 min), roquefortine D (**3**, 18.3 min), glandicoline B (**6**, 18.4 min), meleagrin (**7**, 19.6 min), roquefortine C (**4**, 21.4 min).

In order to identify secondary metabolites synthesized by RoqA, its gene was deleted and comparative metabolite profiling was performed on the culture supernatant of the host and deletion strain. RoqA is specified by a 7.45 kbp long gene that translates into synthetase (2372 amino acids) with the typical domain motifs of nonribosomal peptide synthetases (NRPS). RoqA comprises two adenylation (A), thiolation (T) and condensation (C) domains arranged as ATCATC (Figure 1A) [21]. A host strain *P. chrysogenum* DS54555 was used which derived from a DS17690 strain lacking the *Ku70* gene and thus competent for homologous recombination. The DS54555 strain lacks all penicillin biosynthetic genes clusters, which facilitates the identification of unknown secondary metabolites in the culture broth as the profile is no longer dominated by β-lactam compounds. *RoqA* was deleted by homologous recombination using the deletion plasmid pDEST R4-R3a (see Figure S1A available online) containing the phleomycin resistance gene surrounded by flanking regions of *roqA*. The deletion of *roqA* was confirmed by southern blot hybridization (Figure 2A). The host and Δ*roqA* strain were grown for 168 hours in secondary metabolite production medium (SMP Medium) and comparative metabolite analysis of these strains was carried out by HPLC-UV-MS (Figure S2). This revealed the loss of several secondary metabolites in the deletion strain as compared to the host strain. These metabolites were identified as histidyltryptophanyldiketopiperazine (HTD) (**1**), dehydrohistidyltryptophanyldiketo-piperazine (DHTD) (**2**), roquefortine D (**3**), roquefortine C (**4**), glandicoline B (**6**) and meleagrin (**7**). The structures of all compounds, except **1** and **4** were confirmed by LC-MS/MS analysis and subsequent fragmentation spectra, by retention time comparison with their extracted standards as well as by NMR (Figure 3, Figures S3, S4, S5 and S6, Table S1). The m/z value for the compound **1** identified as HTD was observed at 324.144 dalton representing the protonated molecule MH^+^ with formula C_17_H_18_N_5_O_2_. Its chemical structure was elucidated using LC-MS/MS and high-resolution multi-dimensional fragmentation tree comparison (in preparation). In addition, a protonated molecule MH^+^ at m/z 404.171 and formula C_22_H_22_N_5_O_3,_ which coincides with the protonated form of glandicoline A (**5**), was observed to be present in the host strain but absent in the deletion strain. Although, a full structure elucidation could not be performed, parts of the structure were identified by LC-MS/MS which indicated consistency with the chemical structure of glandicoline A (e.g. dimethylallyl group) (Figure S3E). Hence, this ion was assumed to correspond to protonated glandicoline A. In conclusion, the loss of the identified secondary metabolites in the deletion strain proves unequivocally that RoqA is responsible for the initial reaction in the roquefortine/meleagrin pathway by combining L-tryptophan and L-histidine to **1**.

### Biochemical Analysis of *P. chrysogenum* Harboring a Deletion of the *roqR* Gene

**Figure 4:**
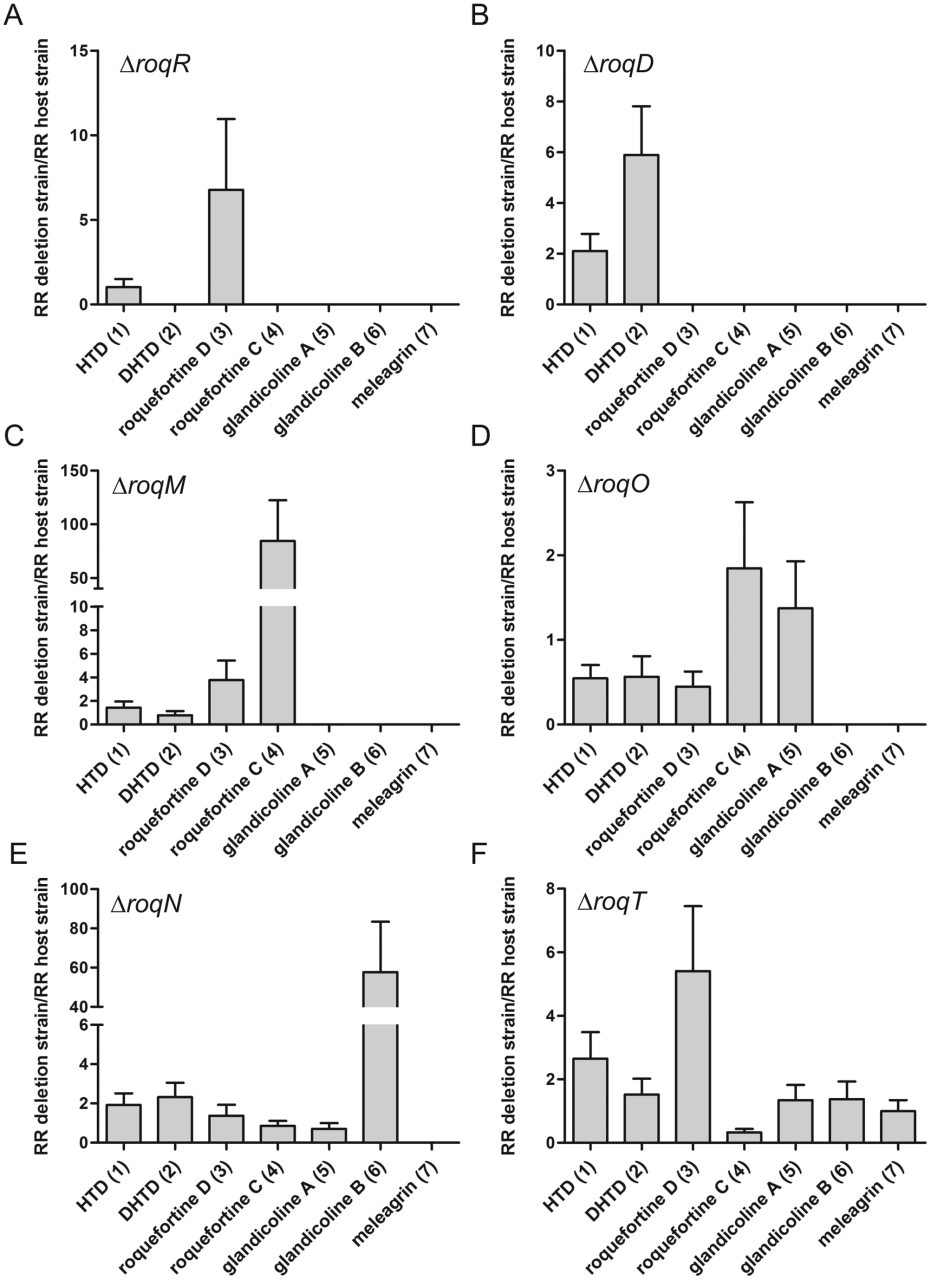
Internal standard corrected concentrations (RR = response ratio) of secondary metabolites from roquefortine/meleagrin pathway. The metabolite concentrations in the culture broth of the Δ*roqR* (A), Δ*roqD* (B), Δ*roqM* (C), Δ*roqO* (D), Δ*roqN* (E) and Δ*roqT* (F) strains was compared to the host strain *P. chrysogenum* DS54555.

In order to elaborate the putative associated genes of the roquefortine/meleagrin biosynthetic pathway, the *roqR* gene encoding a putative cytochrome P450 oxidoreductase was deleted. Herein, a linearized deletion plasmid pDEST R4-R3r (Figure S1B) was transformed into the protoplast of the host strain using the *Aspergillus nidulans* acetamidase gene (*amdS*) for the positive selection of transformants growing on media with acetamide as a sole nitrogen source. The homologous recombination resulted in the complete deletion of the *roqR* gene as demonstrated by Southern blot hybridization (Figure 2B). Both the host and Δ*roqR* strains were grown as described above. Deletion of *roqR* resulted in an accumulation of compound **1** and **3** while **2**, **4**, **5**, **6** and **7** were completely absent in the HPLC-MS profile of the Δ*roqR* strain (Figure 4A). These data suggest that **1** is a precursor of **2** resulting in a branch of the initially proposed linear pathway.

### Biochemical Analysis of *P. chrysogenum* with a Deletion of the *roqD* Gene

RoqD shares high sequence homology with dimethylallyltryptophan synthetase (DMATs). Transcriptional analysis of *roqD* showed high expression in the host strain grown in the absence of PAA in shake flasks (Figure 1B). To examine the role of RoqD in the biosynthesis of roquefortine and related metabolites, its gene was deleted with plasmid pDEST R4-R3d (Figure S1C) using phleomycin as a selection marker (Figure 2C). The deletion strain accumulated high levels of **1** (Figure 4B) in the culture broth as it was unable to add the dimethylallyl group needed for the conversion from **1** to **3**. Moreover, the biosynthesis of the other (downstream) metabolites **3** until **7** did not occur, while **2**, which is similar in structure to compound **1**, was six times higher in concentration as compared to the host strain. In combination with the *roqR* gene deletion, we conclude that RoqD is responsible for the conversion of **1** to **3** (see Discussion).

### Biochemical Analysis of *P. chrysogenum* Strains with a Deletion of *roqM, roqN* and *roqO* Gene

RoqM shows homology to MAK1-like monooxygenases. Its gene was deleted with the deletion plasmid pDEST R4-R3m (Figure S1D) using *amdS* as selection marker (Figure 2D). The deletion of *roqM* led to substantial accumulation of **3** and **4** compared to the host strain whereas (downstream) metabolites like **5** till **7** were not detected (Figure 4C). This demonstrates that *roqM* is involved in the synthesis of **5** using **4**.

RoqO shows homology to cytochrome P450 monooxygenases, and its gene was deleted using plasmid pDEST R4-R3o (Figure 2E, Figure S1E) using *amdS* as selection marker. Metabolite profile comparison of the host and deletion strain showed similar concentrations of **5** and its upstream metabolites in both strains (Figure 4D). The absence of **6** and **7** in the deletion strain suggests **5** as final product and therefore indicates a role of *roqO* in the biosynthesis of **5** to **6**.

*RoqN* specifies a putative methyltransferase. pDEST R4-R3n (Figure S1F) was used for the deletion of *roqN* using *amdS* for the positive selection of transformants (Figure 2F). The deletion of *roqN* resulted in the loss of **7** in fermentation broth (Figure 4E). In addition, the concentration of **6** was increased sixty times compared to the host strain. This increase confirms that RoqN is responsible for the conversion of **6** to **7** [31].

### Biochemical Analysis of *P. chrysogenum* Harboring a Deletion of the *roqT* Gene

RoqT shows high sequence homology with transporters of the major facilitator superfamily. The gene was removed from the genome (Figure 2G) using deletion plasmid pDEST R4-R3t (Figure S1G) and phleomycin as selection marker. Remarkably, the strain with a deletion of *roqT* did not show any marked changes in the metabolite profile as compared to host strain (Figure 4F), except that the production of **3** was increased five times whereas production of **4** was decreased by 60%. This indicates that *roqT* is not essential for the biosynthesis of **1** till **7**.

### Gene Expression and Secondary Metabolite Production

To relate the extracellular metabolites of the roquefortine/meleagrin pathway to the expression levels of the biosynthetic genes, the host strain was grown for 216 hours in secondary SMP medium. Total mRNA extraction and extracellular metabolites analysis were carried with samples collected during growth. The metabolite concentrations were determined by HPLC-UV-MS, while transcript levels were determined by quantitative PCR using γ-actin as reference gene (Figure 1C, Figures S7 and S8). The expression levels of the various biosynthetic genes increased linear in time, except for *roqA* that was found to be highly up-regulated after 96 hours when the cells are in the late log phase. The concentration of metabolite **1** synthesized by RoqA increased almost equally with the expression levels of *roqA* (Figure 1C), while after 192 hours the concentrations of metabolites **5** till **7** in the media decreased (Figure S7 and S8). The concentration of **4** dropped significantly already after 168 hours. The production of all metabolites was particularly high after 96 hours of growth. It is concluded that the production of these metabolites is a late event during growth and that it correlates with the expression of the respective biosynthetic genes.

### Precursor Feed Stimulates Metabolite Production

**Figure 5:**
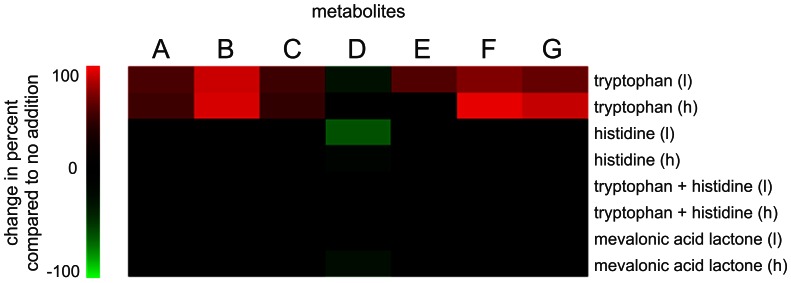
Change in production of roquefortine/meleagrin metabolites after precursor addition compared to production in cultures without addition. Colored cells show mean levels that are significant (P<0.05) different.

To evaluate the role of the predicted precursors in roquefortine/meleagrin pathway and related metabolites production, feeding experiments were performed. L-Tryptophan, L-histidine and mevalonic acid lactone were added individually and in combination (at high and low concentration, i.e., 30 and 10 mM respectively) to the cultures. Since the production of **7** and most of its derivatives is most significant around 96 hours of growth, precursors were added at that time point. Filtered supernatants were analyzed on HPLC-UV-MS after 168 hours of growth. The metabolites concentrations were dry weight corrected and compared via t-test to samples without precursors addition. The concentration of metabolites **1** till **7**, except **4** increased with the addition of 10 mM tryptophan (Figure 5). Addition of 30 mM tryptophan increased the production of **1**, **2**, **3**, **5** and **7** while addition of histidine, mevalonic acid lactone and combinations of tryptophan and histidine did not reveal any significant increase. These data suggest that the roquefortine biosynthetic pathway is limited by the availability of tryptophan.

## Discussion

Here we have elucidated the biosynthetic pathway of *P. chrysogenum* responsible for the production of roquefortine, meleagrin and related compounds. Through a combined metabolic profiling, MS- and NMR based structure elucidation and gene inactivation analysis (7 genes in total, including a transporter-encoding gene); individual genes could be assigned to the various biosynthetic steps. The architecturally complex roquefortine and meleagrin scaffolds are synthesized from simple building blocks, i.e. histidine and tryptophan.

**Figure 6:**
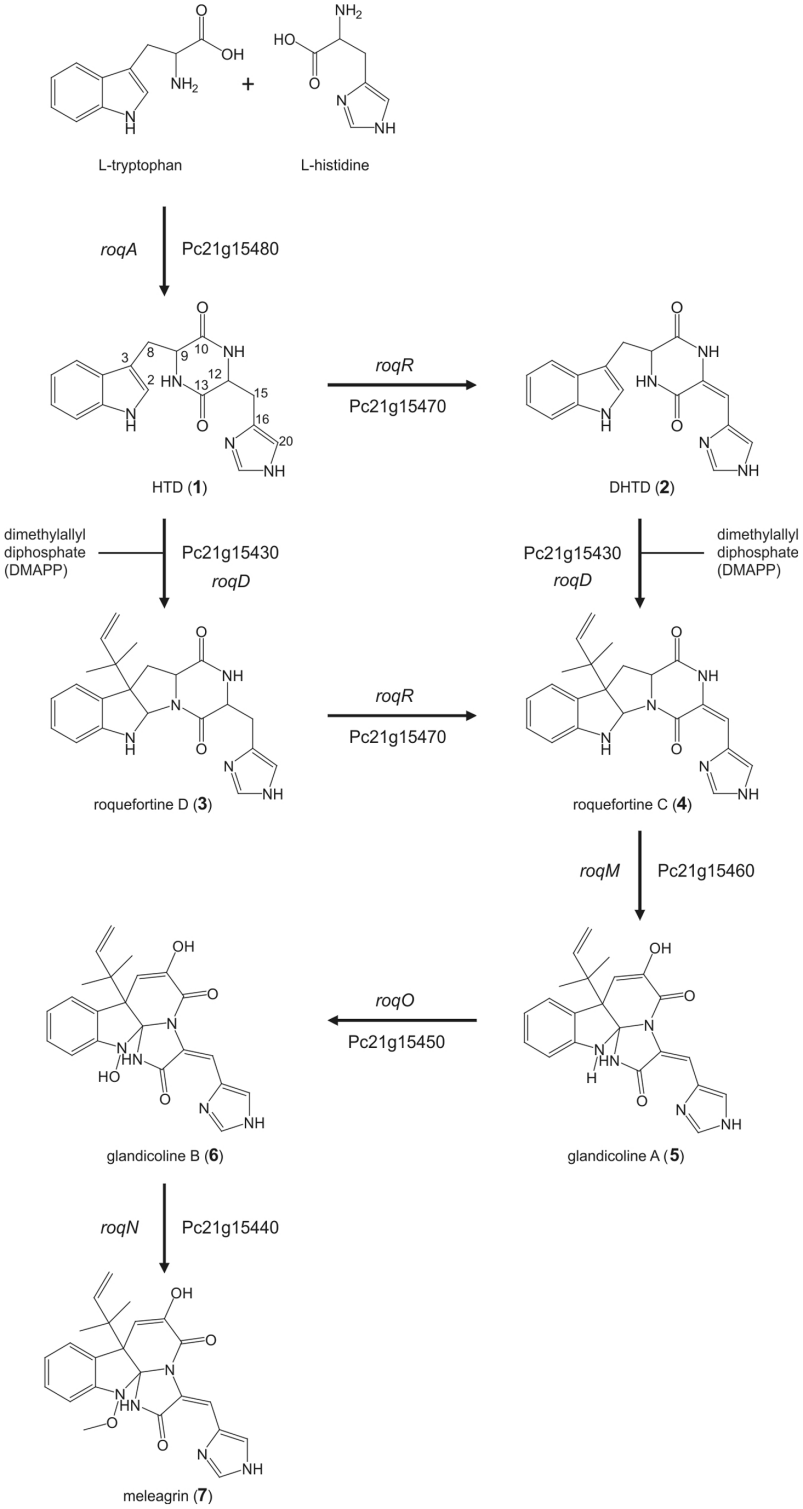
Proposed roquefortine/meleagrin biosynthetic pathway. See text for details.

RoqA is a di-modulated NRPS, containing two adenylation domains [21] responsible for the condensation of tryptophan and histidine to produce **1**, a diketopiperazine. The *roqA* deletion strain no longer produced any of the roquefortine related metabolites (**1** till **7** absent from the broth) which was also recognized by García-Estrada et al. [31]. Therefore, *roqA* encodes the core enzyme initiating the biosynthesis of **1** till **7** and it is present in all species reported to produce these compounds [12], [33]. *RoqD* encodes a dimethylallyltryptophan synthase catalyzing the reversed prenylation of **1** at the C3 position in its indole moiety utilizing dimethylallyl diphosphate derived from mevalonic acid lactone of the mevalonate pathway to form **3** (Figure 6). In addition, it also catalyzes the ring closure between C2 and N14 of the diketopiperazine moiety [34]. Both conversions seem to occur simultaneously, similar to the formation of aszonalenin from benzodiazepinedione, as no intermediate could be observed [35]. No prenylated cyclo-histidyltryptophanyl-diketopiperazine was detected in both the host and deletion strain in contrast to what has previously suggested [31]. The latter we attribute to a lack of convincing analytical evidence that indeed this compound is formed. Importantly, this resulted in a wrong assignment of RoqD and RoqM in the biosynthetic pathway. Reduction of **1** at position C12– C15 of the histidinyl moiety to **2** is carried out by the cytochrome p450 oxidoreductase encoded by *roqR* leading to a previously unknown branch in the pathway. In other studies, **2** was reported as a degradation product of **4** under acidic conditions and in-vivo [17], [36]. However, the accumulation of **2** in the Δ*roqD* strain, despite the absence of **4** unequivocally indicates a different origin for the production of **2**. Two possible reactions of **1** lead to a branch of the roquefortine/meleagrin pathway, one via oxidation by RoqR to **2** and further to **4** by dimethylallyl addition by RoqD, and the other via dimethylallyl addition by RoqD to **3** and further to **4** via oxidation carried out by RoqR (Figure 6). **2** is therefore not just a degradation product of **4** but a true biological product of the roquefortine/meleagrin pathway. The absence of **3** and **4** in the *roqD* deletion strain and the absence of **2** and **4** in the *roqR* deletion strain strongly support our proposal that **2** is the precursor of **4** following an isoprene addition by RoqD at **2** to form **4**, similar to the known reaction of **1** to **3** (Figure 6). These observations are very intriguing as it questions the precursor role of **3** to **4** in the proposed roquefortine/meleagrin pathway [34]. **3** could be the end of the branch leaving **2** as single precursor for **4** if RoqD is unable of isoprene addition on **2** to yield **3**. Therefore, **2** or **3** or even both can be possible precursors of **4**.

Based on the highly significant accumulation of **4** in the *roqM* deletion strain and the absence of all downstream metabolites **5** till **7**, we concluded that *roqM*, encoding a MAK 1-monooxygenase like protein, is involved in the conversion of **4** to **5**. This leads to the conclusion that only one enzyme is involved in this prominent conversion which combines an oxidation, ring opening and ring closure in one reaction cascade [37]. A possible mechanism involves an initial hydroxylation at C9 with a subsequent cleavage of the bond between C9 and N14, yielding a nine-membered ring with a keto-group at C9. Further oxidation at N1 of the indole moiety could lead to the loss of water to generate an imine which is followed by an attack of N11, ultimately yielding glandicoline A. Maackiain monooxygenases are known to be responsible for the minor conversion of Maackiain to 1-Hydroxymackian by the addition of an oxygen, which differs from the more prominent reaction from **4** to **5** found here [38]. It should however be emphasized that the enzyme assignment was initially based on sequence alignment only. Further definition of the mechanism requires an enzymological characterization.

Our results also demonstrate an involvement of RoqO in the conversion of **5** to **6**. *roqO* encodes a p450 monooxygenase that oxidizes **5** on its tryptophan moiety, resulting in formation of **6**. Remarkably, García-Estrada et al. [31]mixed up the reactions catalyzed by RoqO and RoqM most likely because the lower concentrations of **6** were not recognized, whereas **5** could not have been detected at all with the used HPLC-UV approach. Therefore, these authors also wrongly assigned the biosynthetic pathway leading to the production of **5** by RoqO and **6** by RoqM. RoqN shares 99% identity with the UbiE/COQ5 family methyltransferase in *A. origami.* Its activity was confirmed by inactivation of the *roqN* gene resulting in a loss of **7** concomitantly with a 60 times increase of **6.** Thus **7** is the methylated form of **6**, consistent with an involvement of methyltransferase as earlier recognized [31].

Finally, *roqT* encodes a highly expressed member of the Multifacilitator Super Family of transporters that might be involved in active transport of some of these metabolites. However, its deletion had little effect on the metabolic profile suggesting that passive transport, diffusion or another transporter might be involved in secretion. It is remarkable that all of the described intermediates are found in the extracellular broth which suggests that these compounds effectively diffuse from the cell. Alternatively, secretion might be mediated by a relative unspecific export system that has not been identified so far. RoqT might fulfill a role in the retention of these metabolites (see also below) but this needs to be investigated further in feeding experiments.

In order to explore the evolutionary context of this gene cluster among filamentous fungi the roquefortine/meleagrin gene clusters in various fungi were compared using the antiSMASH algorithm [32]. Sorting the gene clusters by an empirical similarity score based on the number of BlastP hits between the predicted proteins of the gene clusters, gene order conservation and the percentage identity of the Blast hits resulted in three hits of gene clusters carrying more than two homologs of genes from the roquefortine/meleagrin biosynthesis gene cluster. One gene cluster, encoded in the genome of *Neosartorya fisheria*, contained three genes closely similar to genes of the *P. chrysogenum* roquefortine/meleagrin biosynthetic pathway (59, 67 and 60% identity with *roqA*, *roqD* and *roqO*, respectively) (Figure 1A, Table S2). This suggests that *N. fisheria* may be capable of synthesizing compounds similar to **1, 2, 3** and **4**, using similar biosynthetic mechanisms. *A. fumigatus* and *A. terreus* also have three genes in common with the *P. chrysogenum* roquefortine gene cluster (*roqA*, *roqD* and *roqO*). Metabolites produced by these organisms are likely similar to **1** and **3**. However, the final structures may be different as for instance *A. fumigatus* contains at least four additional genes (annotated as phytanoyl-CoA dioxygenase, cytochrome P450, O-methyltransferase and cytochrome P450 monooxygenase) that might be involved in further modifications of these secondary metabolites (Table S2).

A time scale study for the production of roquefortine/meleagrin biosynthesis pathway intermediates and products showed an accumulation of **7** in the fermentation broth over time. After 192 hours of growth the metabolite concentrations reached their maximum before it declined due to a possible uptake from the media or degradation. Uptake of intermediates back into mycelia was previously reported for roquefortine [39] and it was proposed that these compounds serve as exogenous nitrogen source for colonial expansion [18]. As tryptophan, histidine and mevalonic acid lactone are the building blocks of the roquefortine/meleagrin pathway their effect on the production of **1–7** was determined in feeding experiments. Although labeling experiments showed the uptake and incorporation of these precursors [40] only the presence of tryptophan stimulated secondary metabolite formation by this pathway (Figure 5). This implies that production is limited by the availability of tryptophan providing a lead for the optimization of roquefortine/meleagrin pathway by metabolic engineering of the shikimic acid or anthranilate route for tryptophan biosynthesis [41]. On the other hand, histidine reversed the effect of tryptophan. This might relate to nitrogen-dependent regulation of gene expression as roquefortine and related compounds have been implicated as exogenous nitrogen sources for colonial expansion [18], and histidine is an excellent nitrogen source for *P. chrysogenum*. Finally, we are currently analyzing additional intermediates for structural elucidation. It is most likely that some of the enzymes of this pathway lack a certain degree of substrate specificity, which may lead to further branching and thus a wider palette of roquefortine/meleagrin related compounds.

### Conclusion

Genome sequencing and microarray data analysis revealed seven genes clustered in the genome of *Penicillium chrysogenum* which are highly up regulated in the absence of the penicillin G precursor phenylacetic acid. These genes are involved in the biosynthesis of roquefortine/meleagrin metabolites. Complete deletion of all seven genes and detailed biochemical analysis of respective mutants strains via HPLC, MS and NMR revealed that dehydrohistidyltryptophanyldiketopiperazine (DHTD), which was previously identified as degradation product, is biologically synthesized and a precursor to Roquefortine C. Two enzymes of this pathway catalyze more than one reaction i.e. RoqD converts histidyltryptophanyldiketopiperazine (HTD) to roquefortine D and DHTD to roquefortine C while RoqR converts HTD to DHTD and roquefortine D to roquefortine C. Thus meleagrin is synthesized via a branched pathway rather than a linear biosynthesis.

## Supporting Information

Figure S1 **Map of the deletion constructs for** ***roqA*** **,** ***roqR*** **,** ***roqD, roqM, roqO, roqN*** **and** ***roqT*** **which were used for deletion.** Features of the vectors: Amp, Ampicillin resistance gene for the selection in *E. coli*; *ori*, pUC origin of replication; attP3, and attP4, Gateway *att* recombination sites; Pipns, promoter promoter of *P. chrysogenum pcbC* gene; Phleomycin, resistance gene for selection in fungi. *amdS*, *A. nidulans* acetamidase gene.
(TIF)

Figure S2 **Analytical approach in schematic view.** Proteins, present in fermentation broth, were removed during sample preparation. Samples were analyzed by HPLC-UV-MS and comparative metabolite profiling performed. Statistical significant features were extracted using liquid-liquid extraction (LLE) and semi-preparative HPLC-UV-MS and their structure elucidated by NMR.
(TIF)

Figure S3 **HPLC-MS/MS fragmentation spectra including chemical formula and calculated exact mass of the protonated HTD (A), DHTD (B), roquefortine D (C), roquefortine C (D), glandicoline A (E), glandicoline B (F) and meleagrin (G) acquired at LTQ-FT-MS Ultra at 35% normalized collision energy in positive mode.**
(TIF)

Figure S4 **^1^H NMR spectrum of DHTD (2).**
(TIF)

Figure S5 **^1^H NMR spectrum of roquefortine D (3).** Small additional peaks are not due to impurities but to a second conformation of roquefortine D.
(TIF)

Figure S6 **^1^H NMR spectrum of glandicoline B (6).**
(TIF)

Figure S7 **Internal standard corrected metabolite concentration in fermentation broth of** ***P. chrysogenum*** **AFF393 sampled at multiple time points and determined by HPLC-UV-MS.**
(TIF)

Figure S8 **Temporal expression of roquefortine/meleagrin biosynthetic gene cluster in** ***P. chrysogenum*** **AFF393 grown in shaking flask culture.**
(TIF)

Table S1 **^1^H and ^13^C NMR chemical shifts of DHTD (2), roquefortine D (3) and glandicoline B (6) in DMSO/CDCl_3_ at 320K and 280K, respectively (δ in ppm).**
(DOCX)

Table S2 **BlastP analysis of roquefortine/meleagrine biosynthetic pathway genes.**
(DOCX)

Table S3 **Primers designed for gene expression analysis of roquefortine/meleagrine biosynthetic gene.**
(DOCX)
