# Supplementary figures and images for: A Branched Biosynthetic Pathway Is Involved in Production of Roquefortine and Related Compounds in Penicillium chrysogenum
Source: PLoS One. 2013 Jun 12;8(6):e65328. doi: 10.1371/journal.pone.0065328 (PMC3680398; doi:10.1371/journal.pone.0065328)

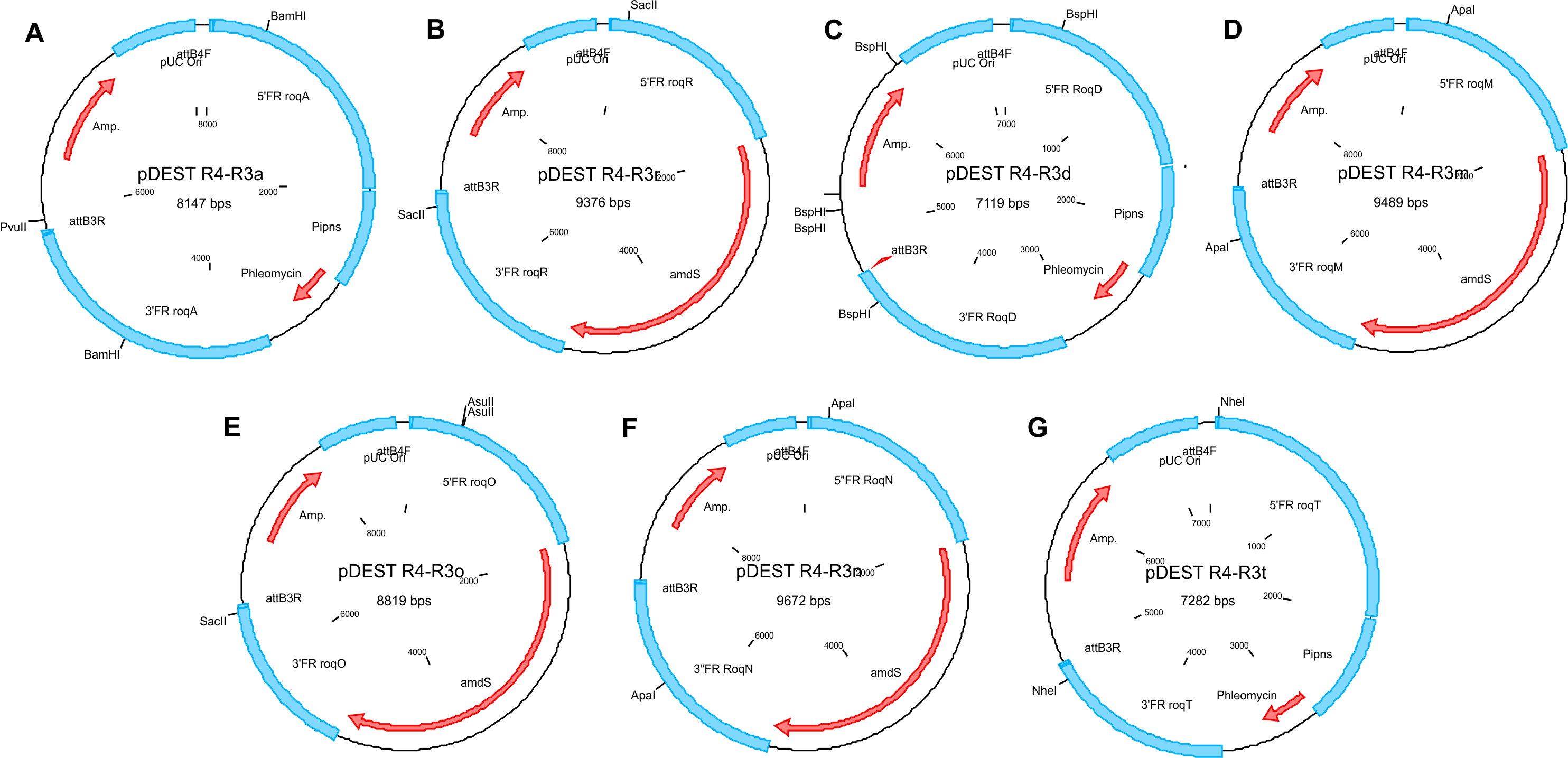

Supplement: Figure S1 — Map of the deletion constructs for roqA , roqR , roqD, roqM, roqO, roqN and roqT which were used for deletion. Features of the vectors: Amp, Ampicillin resistance gene for the selection in E. coli; ori, pUC origin of replication; attP3, and attP4, Gateway att recombination sites; Pipns, promoter promoter of P. chrysogenum pcbC gene; Phleomycin, resistance gene for selection in fungi. amdS, A. nidulans acetamidase gene. (TIF) [file pone.0065328.s001.tif]

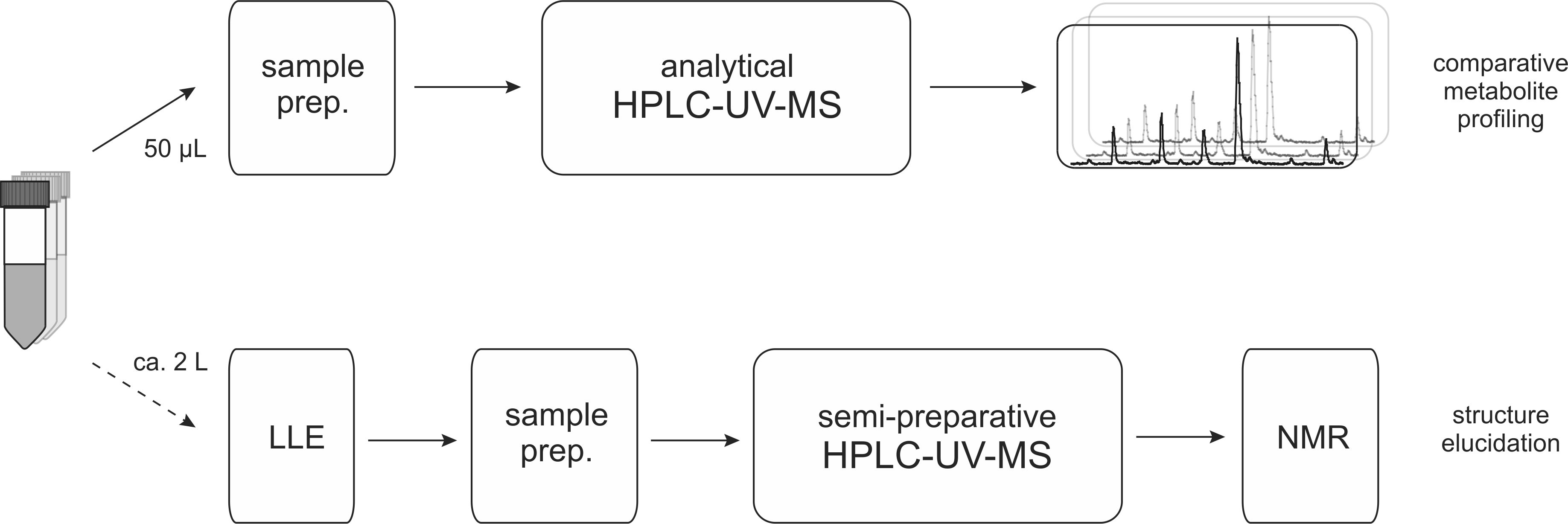

Supplement: Figure S2 — Analytical approach in schematic view. Proteins, present in fermentation broth, were removed during sample preparation. Samples were analyzed by HPLC-UV-MS and comparative metabolite profiling performed. Statistical significant features were extracted using liquid-liquid extraction (LLE) and semi-preparative HPLC-UV-MS and their structure elucidated by NMR. (TIF) [file pone.0065328.s002.tif]

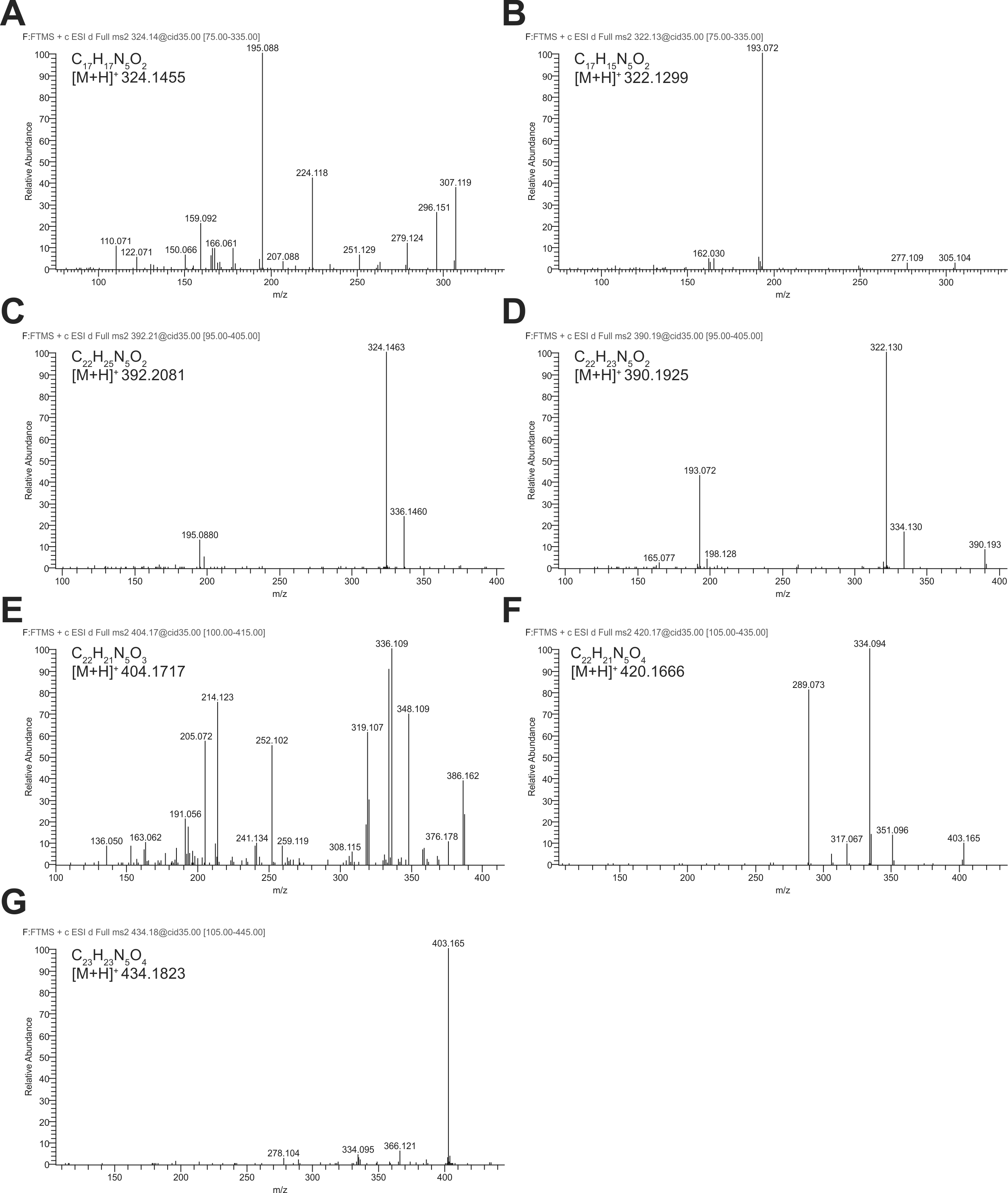

Supplement: Figure S3 — HPLC-MS/MS fragmentation spectra including chemical formula and calculated exact mass of the protonated HTD (A), DHTD (B), roquefortine D (C), roquefortine C (D), glandicoline A (E), glandicoline B (F) and meleagrin (G) acquired at LTQ-FT-MS Ultra at 35% normalized collision energy in positive mode. (TIF) [file pone.0065328.s003.tif]

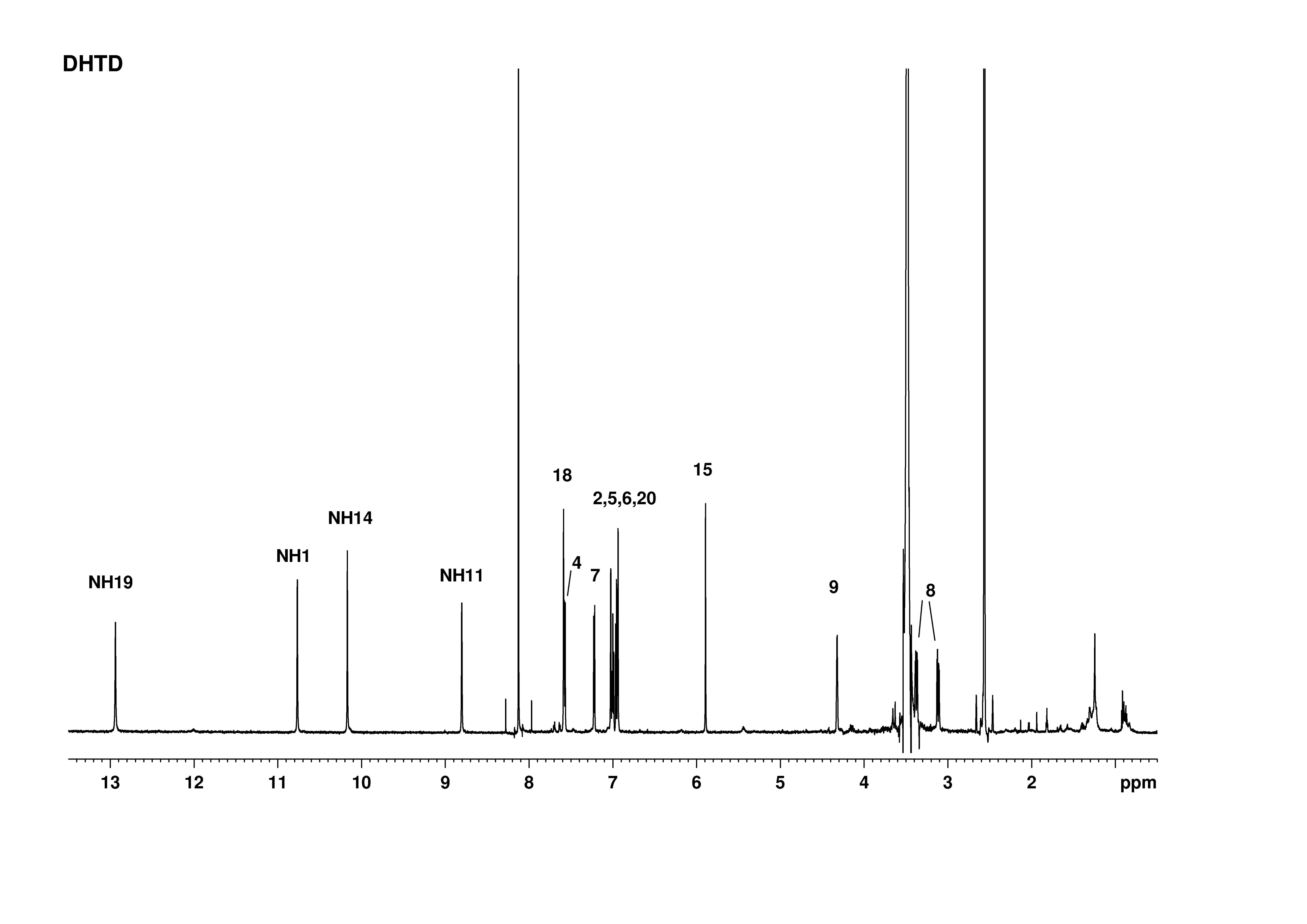

Supplement: Figure S4 — 1H NMR spectrum of DHTD (2). (TIF) [file pone.0065328.s004.tif]

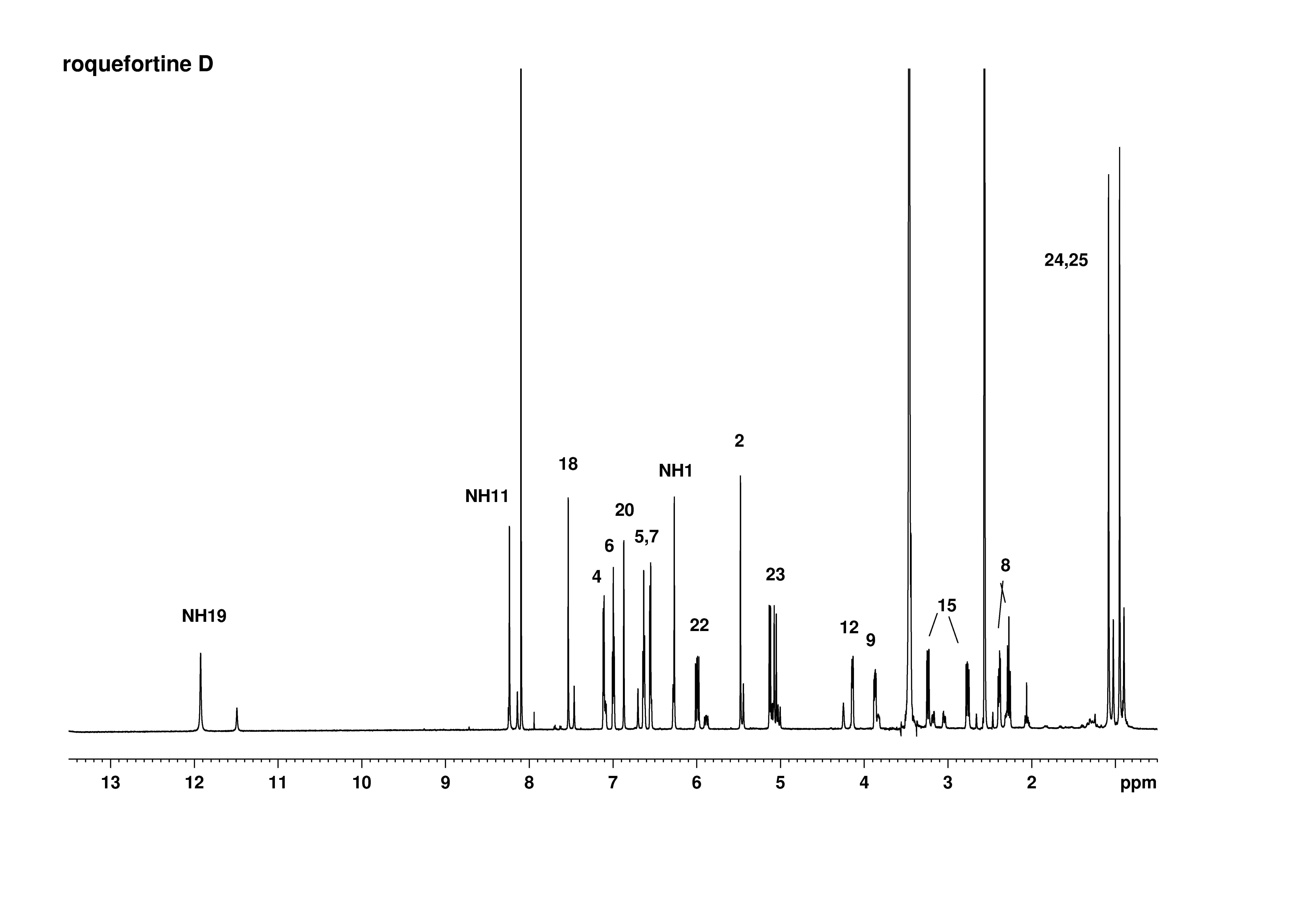

Supplement: Figure S5 — 1H NMR spectrum of roquefortine D (3). Small additional peaks are not due to impurities but to a second conformation of roquefortine D. (TIF) [file pone.0065328.s005.tif]

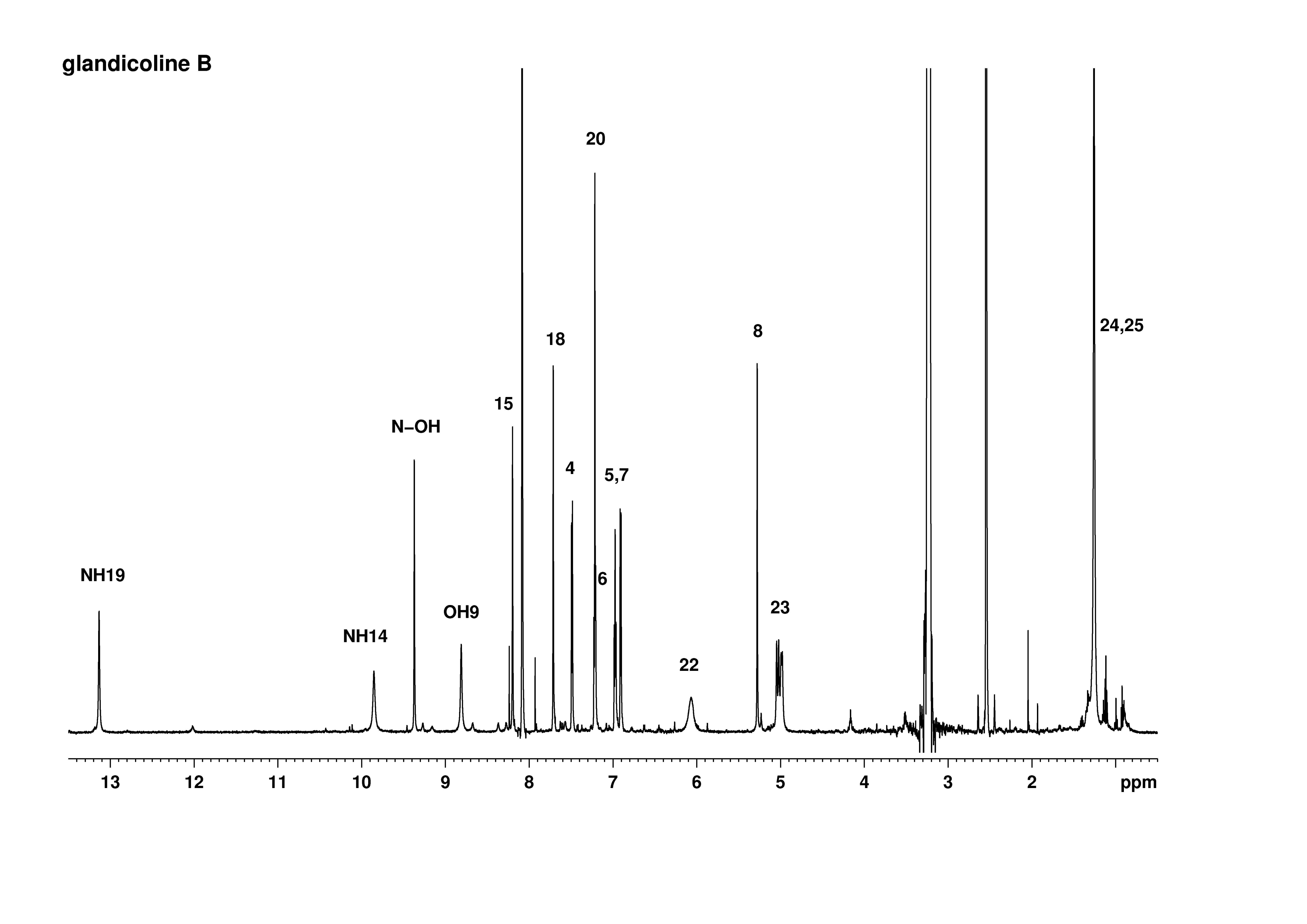

Supplement: Figure S6 — 1H NMR spectrum of glandicoline B (6). (TIF) [file pone.0065328.s006.tif]

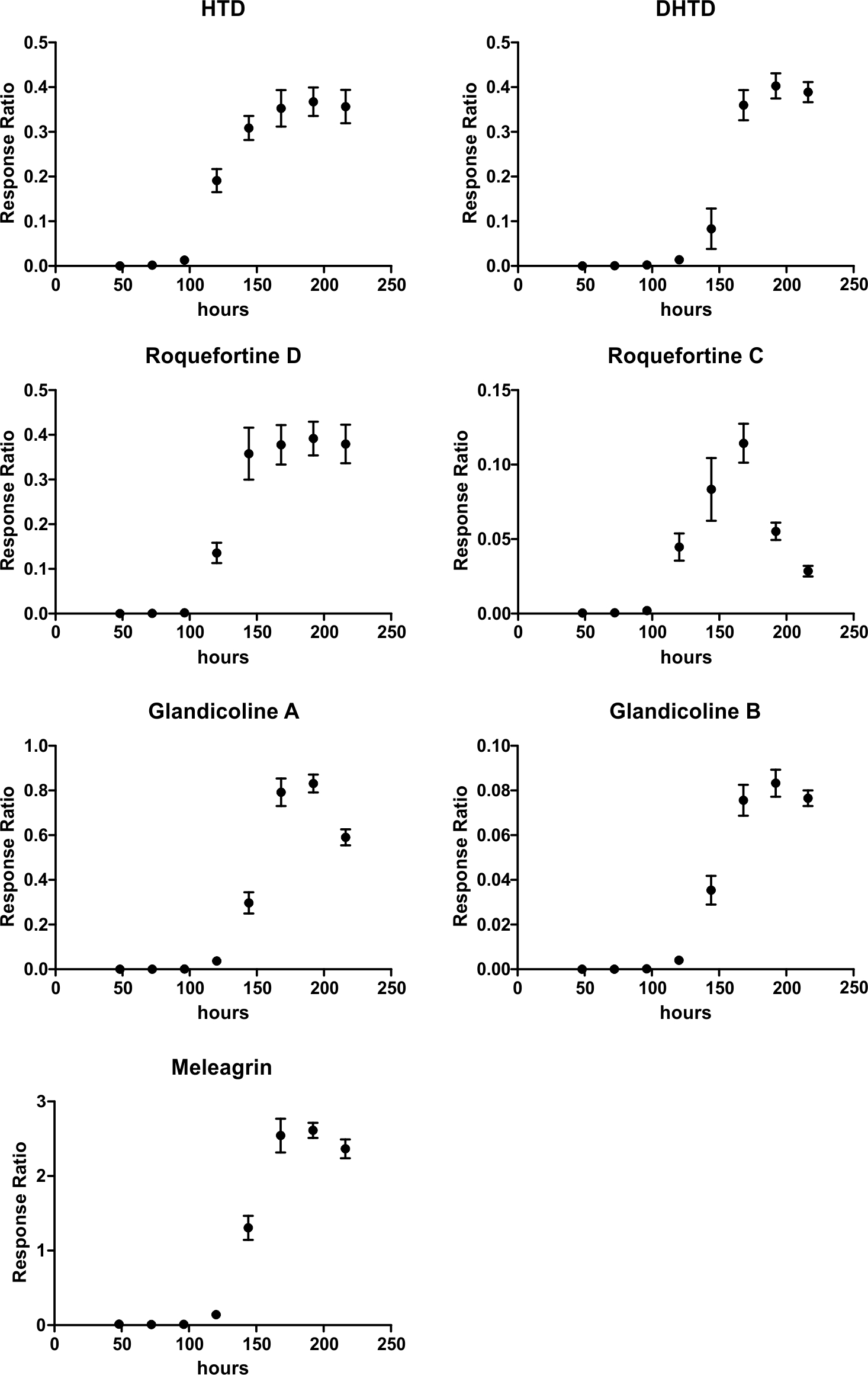

Supplement: Figure S7 — Internal standard corrected metabolite concentration in fermentation broth of P. chrysogenum AFF393 sampled at multiple time points and determined by HPLC-UV-MS. (TIF) [file pone.0065328.s007.tif]

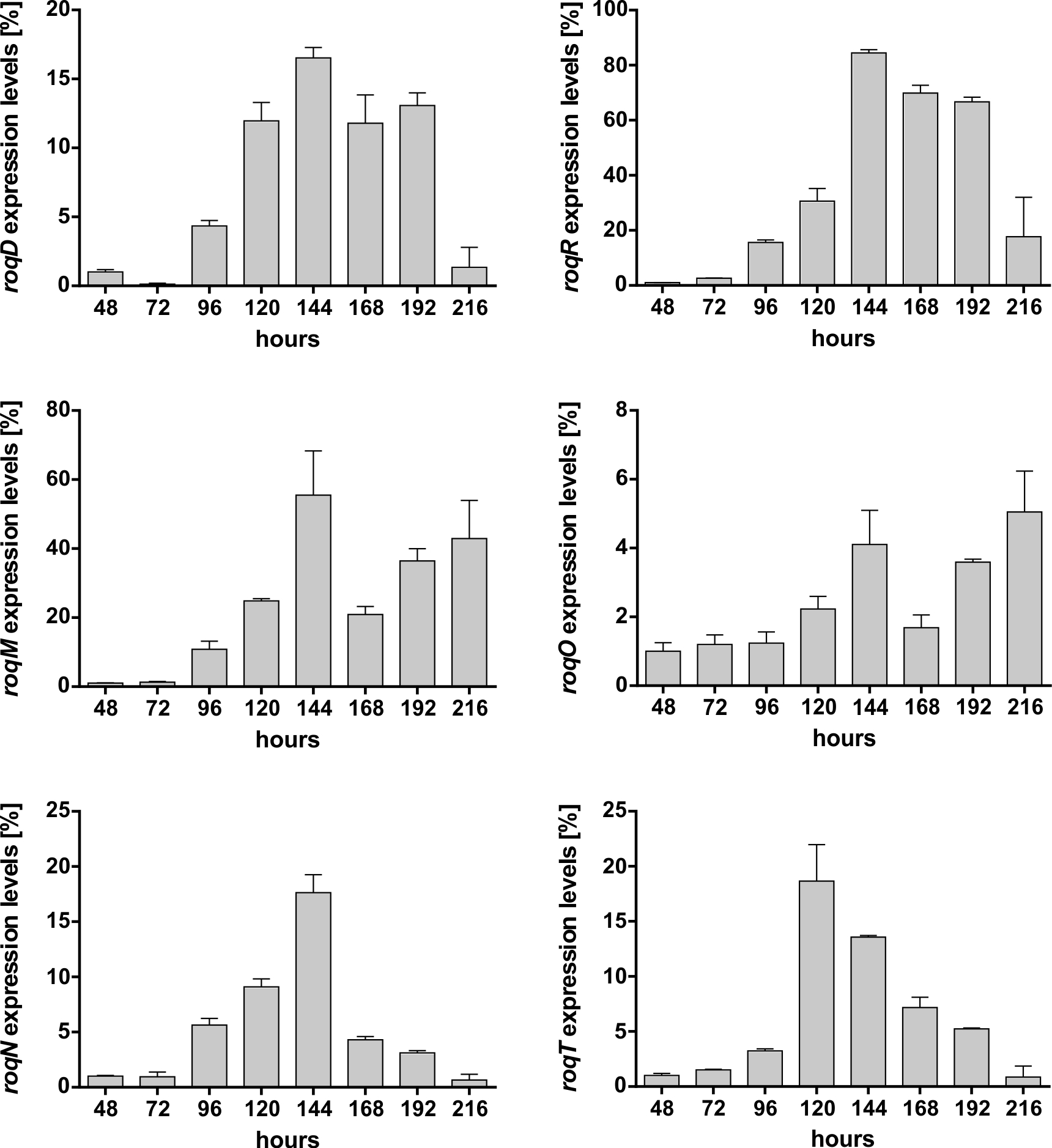

Supplement: Figure S8 — Temporal expression of roquefortine/meleagrin biosynthetic gene cluster in P. chrysogenum AFF393 grown in shaking flask culture. (TIF) [file pone.0065328.s008.tif]
