# Supplementary material for: A Branched Biosynthetic Pathway Is Involved in Production of Roquefortine and Related Compounds in Penicillium chrysogenum
Source: PLoS One. 2013 Jun 12;8(6):e65328. doi: 10.1371/journal.pone.0065328 (PMC3680398; doi:10.1371/journal.pone.0065328)
Supplement: Table S1 — 1H and 13C NMR chemical shifts of DHTD (2), roquefortine D (3) and glandicoline B (6) in DMSO/CDCl3 at 320K and 280K, respectively (δ in ppm). (DOCX) [file pone.0065328.s009.docx]

| DHTD (**2**)  TE=280K | | | roquefortine D (**3**)  TE=280K | | | glandicoline B (**6**)  TE=320K | | |
| --- | --- | --- | --- | --- | --- | --- | --- | --- |
| ^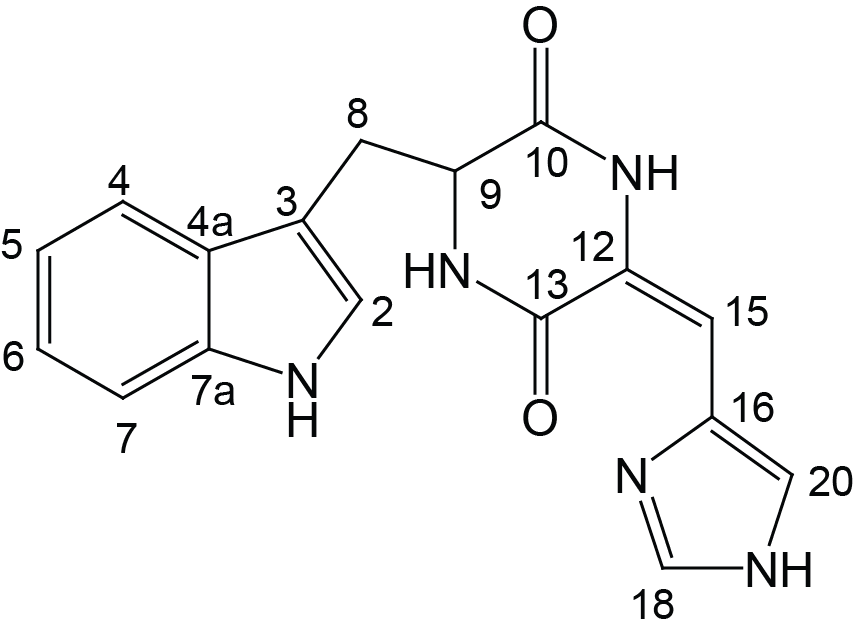^ | | | 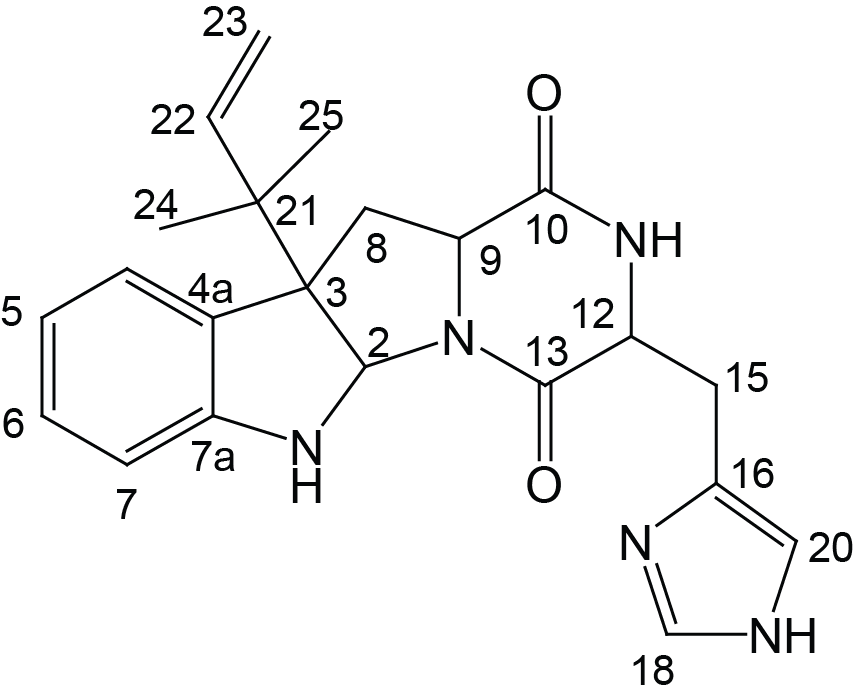 | | | 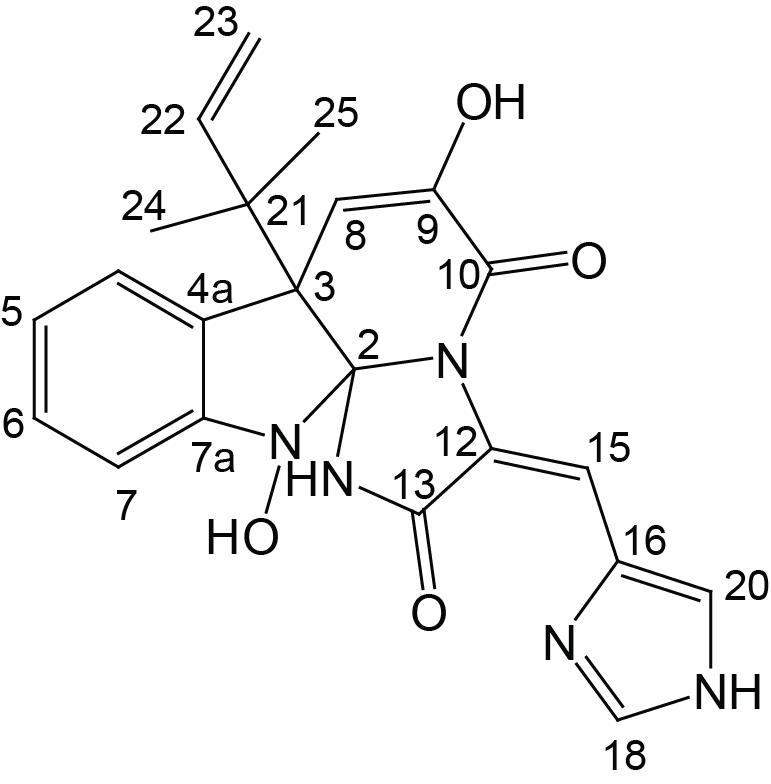 | | |
|  | ^1^H (δ) | ^13^C (δ) |  | ^1^H (δ) | ^13^C (δ) |  | ^1^H (δ) | ^13^C (δ) |
| NH1 | 10.77 | n.a. | NH1 | 6.27 | n.a. | NOH1 | 9.40 | n.a. |
| C2 | 7.03 | 124.4 |  | 5.48 | 76.7 |  | n.a. | 101.7 |
| C3 | n.a. | 106.9 |  | n.a. | 60.7 |  | n.a. | 52.5 |
| C4 | 7.58 | 118.6 |  | 7.12 | 124.5 |  | 7.51 | 124.1 |
| C4a | n.a. | 127.4 |  | n.a. | 128.6 |  | n.a. | 126.5 |
| C5 | 6.96 | 118.4 |  | 6.64 | 117.4 |  | 6.99 | 122.0 |
| C6 | 7.00 | 120.7 |  | 7.00 | 128.3 |  | 7.23 | 127.6 |
| C7 | 7.22 | 111.0 |  | 6.56 | 108.5 |  | 6.93 | 111.4 |
| C7a | n.a. | 136.1 |  | n.a. | 150.8 |  | n.a. | 148.2 |
| C8 | 3.38 3.12 | 29.6 |  | 2.39 2.28 | 36.5 |  | 5.29 | 109.0 |
| C9 | 4.32 | 56.0 |  | 3.87 | 58.2 |  | n.a. | 142.8 |
| OH9 | n.a. | n.a. |  | n.a. | n.a. |  | 8.86 | n.a. |
| C10 | n.a. | 165.8 |  | n.a. | 168.2 |  | n.a. | 158.8 |
| NH11 | 8.81 | n.a. |  | 8.24 | n.a. |  | n.a. | n.a. |
| C12 | n.a. | 121.6 |  | 4.14 | 55.2 |  | n.a. | 124.4 |
| C13 | n.a. | 160.9 |  | n.a. | 164.9 |  | n.a. | 166.5 |
| NH14 | 10.17 | n.a. |  | n.a. | n.a. |  | 9.90 | n.a. |
| C15 | 5.90 | 107.4 |  | 2.77 3.24 | 28.0 |  | 8.20 | 106.8 |
| C16 | n.a. | 125.5 |  | n.a. | 136.3 |  | n.a. | 126.1 |
| NH17 | 12.91 | n.a. |  | 11.93 | n.a. |  | 13.16 | n.a. |
| C18 | 7.59 | 135.8 |  | 7.54 | 134.8 |  | 7.73 | 136.9 |
| C20 | 6.94 | 133.0 |  | 6.87 | 113.3 |  | 7.23 | 133.5 |
| C21 | n.a. | n.a. |  | n.a. | 40.6 |  | n.a. | 41.8 |
| C22 | n.a. | n.a. |  | 6.00 | 143.7 |  | 6.08 | 143.3 |
| C23 | n.a. | n.a. |  | 5.13 5.06 | 113.9 |  | 5.05 5.00 | 112.6 |
| C24 | n.a. | n.a. |  | 1.09 | 22.1 |  | 1.28 | 23.4 |
| C25 | n.a. | n.a. |  | 0.95 | 22.6 |  | 1.28 | 23.4 |
