# Supplementary material for: A Branched Biosynthetic Pathway Is Involved in Production of Roquefortine and Related Compounds in Penicillium chrysogenum
Source: PLoS One. 2013 Jun 12;8(6):e65328. doi: 10.1371/journal.pone.0065328 (PMC3680398; doi:10.1371/journal.pone.0065328)
Supplement: Table S2 — BlastP analysis of roquefortine/meleagrine biosynthetic pathway genes. (DOCX) [file pone.0065328.s010.docx]

| *Neosartorya fischeri* NRRL 181 | | | | | |
| --- | --- | --- | --- | --- | --- |
| **Query gene** | **Subject gene** | **% Identity** | **Blast score** | **%Coverage** | **E-value** |
| *roq D* | NFIA_074280 | 67 | 602 | 100 | 0.0 |
| *roq R* | NFIA_074290 | 60 | 598 | 100 | 0.0 |
| *roq A* | NFIA_074230 | 59 | 2644 | 92 | 0.0 |
| *Aspergillus fumigatus* Af293 | | | | | |
| **Query gene** | **Subject gene** | **% Identity** | **Blast score** | **%Coverage** | **E-value** |
| *roq D* | AFUA_8G00210 | 31 | 206 | 100 | 1e-62 |
| *roq O* | AFUA_8G00240 | 65 | 646 | 94 | 0.0 |
| *roq A* | AFUA_8G00170 | 32 | 955 | 89 | 0.0 |
| *Aspergillus terreus* NIH2624 | | | | | |
| **Query gene** | **Subject gene** | **% Identity** | **Blast score** | **%Coverage** | **E-value** |
| *roq D* | ATEG_10306 | 31 | 226 | 100 | 7e-71 |
| *roq O* | ATEG_10307 | 47 | 333 | 70 | 5e-112 |
| *roq A* | ATEG_10305 | 32 | 920 | 81 | 0.0 |
