# Supplementary material for: A Branched Biosynthetic Pathway Is Involved in Production of Roquefortine and Related Compounds in Penicillium chrysogenum
Source: PLoS One. 2013 Jun 12;8(6):e65328. doi: 10.1371/journal.pone.0065328 (PMC3680398; doi:10.1371/journal.pone.0065328)
Supplement: Table S3 — Primers designed for gene expression analysis of roquefortine/meleagrine biosynthetic gene. (DOCX) [file pone.0065328.s011.docx]

| Target | Primer sequence (5’- 3’) | | |
| --- | --- | --- | --- |
|  | Forward | Reverse | |
| *roqA* | AATTAGTGGCTTCATCTCC | | CGGGTGATATACTGCAGTCC |
| *roqD* | CTTGGTCGGCATTCCCGAGC | | ATAGTACATGGTGAGGTATGG |
| *roqR* | CCTGCGCAATACACTGGCGG | | TGACACGGCTCCTGAATCATGG |
| *roqM* | CTCGCATCTGACTATAAATCGC | | CTCGCAGACTACAAGATCATC |
| *roqO* | GACGACGATTGCTGACACC | | CATGGTTATGCAGCGAGCC |
| *roqN* | CAGTCCACTCCTGTGGCACC | | GAATTCATGTCCTGTATGAACC |
| *roqT* | AACTGATCCTCTACCGCAGG | | GTGAGTCGAACTGATCTGTG |
